# The Application of the Materia Medica

**Published:** 1896-01

**Authors:** C. M. Boger

**Affiliations:** Parkersburg, W. Va.


### THE APPLICATION OF THE MATERIA MEDICA.

C. M. Boger, M. D., Parkersburg, W. Va.

The method of selecting the truly homœopathic (curative) remedy is a subject demanding the physician's best thought and discrimination. During the course of many of the ordinary, especially acute diseases, the remedy frequently stands out so plainly that "He that runs may read." But every prescriber will daily meet cases that look anything but clear, and require study and reference. In the latter instance, having carefully taken *all* the symptoms, it is essential that the physician be able to pick from among their number, these, with their modalities, which characterize and individualize that particular case. If incompetent to do this, all further progress is at an end. Successful here, the next step is to find these selected symptoms in the *Materia Medica*. For this a good repertory *may* come into play, but nearly all such works are sadly disappointing if depended upon absolutely, and at best are only finger-boards giving us the general direction to be followed, necessitating a final reference to the original text. Having found the remedies possessing one (or all) of the selected symptoms, we exclude those lacking the proper modality of the case in hand ; this brings the selection very near or quite home.

At first sight this may seem a very easy process, but, in fact, it is quite the reverse. In the first place, to be able to quickly select the distinguishing symptom requires an intimate knowledge of all the symptoms common to any given disease in order that the uncommon ones may be readily discerned. Then the finding of this uncommon symptom in the *Materia Medica* takes time and perseverance, and above all, the ability to see it when found, for there are those that have eyes and are blind and ears yet hear nothing. The disease-picture presents to them no central focus around which are grouped, in a more or less orderly manner all the symptom groups which complete and round it out.

We see it then remains largely a matter of discerning the key-symptom, of recognizing the remedy then before you, and proper judgment in its administration.

A certain patient had, among many symptoms, a hiccough, coming on with the first mouthful swallowed, but ceasing on continuing to eat ; this greatly alarmed her, as her father's fatal illness had begun in a precisely similar manner. Naturally Rhus came to mind, but a reference to the proving failed to find that symptom. I now took up the remedies *seriatim*. Bovista had a similar symptom ; then came Conium. As soon as I discovered the symptom under this remedy its general homœopathicity was unmistakable ; and why ? You have never met John Smith, but you know his brother William. Some day you will meet a stranger. One look is enough. You don't need a treatise on physiognomy to know him as William's brother. Conium$^{45m}$, one dose, cured that hiccough and unraveled the knotted skein of symptoms, making the next remedy plain.

A young lady with numerous symptoms had a neuralgia, waking her earlier every morning. Magnesia-phos. quickly palliated, but in a week it returned with redoubled force. Mag-carb. cured it more slowly, but permanently. This brings me to the concluding thought, *i. e.*, after all, your key-symptom will generally be most expeditiously found by taking down the *Materia Medica* and searching diligently and thoroughly, not to mention the many good friends you will learn to know in this way only ; leaving, after all, much less time to grumble about chaff, don't you know.

